# Randomized placebo-controlled, double-blind clinical trial of nanoemulsion curcumin in women with aromatase inhibitor-induced arthropathy: an Alliance/NCORP pilot trial

**DOI:** 10.1007/s10549-023-07223-4

**Published:** 2024-01-27

**Authors:** Maryam Lustberg, Patty Fan-Havard, F. Lennie Wong, Kasey Hill, Mitch A. Phelps, Kevin W. Herrera, Ni-Chun Tsai, Timothy Synold, Ye Feng, Chidimma Kalu, Mina S. Sedrak, Lisa D. Yee

**Affiliations:** 1 Yale Cancer Center, New Haven, CT, USA; 2 Kaweah Health Care District, Visalia, CA, USA; 3 City of Hope Comprehensive Cancer Center, Duarte, CA, USA; 4 Comprehensive Cancer Center, The Ohio State University Wexner Medical Center and James Cancer Hospital, Columbus, OH, USA; 5 David Geffen School of Medicine, University of California, Los Angeles, Los Angeles, USA; 6 Department of Surgery, City of Hope Comprehensive Cancer Center, 1500 East Duarte Road, Duarte, CA 91010, USA

**Keywords:** Nanoemulsion curcumin, Aromatase inhibitors-induced joint arthropathy, Postmenopausal women, Plasma curcumin levels, Plasma estrone and estradiol levels

## Abstract

**Purpose:**

Aromatase inhibitor (AI) therapy reduces risk of recurrence and death for postmenopausal women with breast cancer (BC); however, AI-induced arthralgia (AIIA) can lead to discontinuation of treatment. Curcumin, a bioactive polyphenolic substance, may help ameliorate inflammation-related conditions including osteoarthritis and pain.

**Methods:**

We conducted a multisite randomized placebo-controlled, double-blind pilot trial (Alliance A22_Pilot9) to evaluate the effects of nanoemulsion curcumin (NEC, 200 mg/day) in postmenopausal women experiencing AIIA for ≥ 3 months. The primary objective was to determine the feasibility of using Functional Assessment of Cancer Treatment-Endocrine Symptoms (FACT-ES) to detect changes from 0 (T0) to 3 months (T3) of NEC treatment in AI-induced symptoms and well-being; secondary objectives included evaluation of changes in Disabilities of the Shoulder, Arm, and Hand (DASH), Brief Pain Inventory-short form (BPI-SF), grip strength, and biomarkers at T0 and T3.

**Results:**

Forty-two patients were randomized to NEC or placebo; 34 women completed the 3-month study. Patient-reported outcome measures (PROMs: FACT-ES, DASH, BPI-SF) and biospecimens were collected at T0-T3 in > 80% of participants. Adherence was ≥ 90% for both arms. PROMs and grip strength did not differ significantly by treatment arm. Plasma curcumin was detected only in NEC arm participants. Serum estradiol and estrone levels were below detection or low on study agent. Gastrointestinal adverse effects were commonly reported in both arms.

**Conclusion:**

NEC versus placebo in a multisite randomized trial is feasible and well-tolerated. Additional studies with larger sample size are needed to further evaluate the efficacy and safety of NEC in treatment of AIIA.

ClinicalTrials.gov Identifier: NCT03865992, first posted March 7, 2019.

## Introduction

Aromatase inhibitor (AI) therapy reduces risk of recurrence and death from estrogen receptor (ER)- and/or progesterone receptor (PR)-positive breast cancer (BC). By preventing peripheral conversion of tissues such as fat to estrogen in postmenopausal women, AIs cause profound estrogen deprivation. Recent studies also indicate benefits of prolonged therapy with these drugs for 7–10 years in patients with higher risk BC[1–3].

Despite the efficacy of AI therapy for postmenopausal ER-positive BC, side effects may prove limiting. Musculoskeletal symptoms of joint pain and stiffness affect up to 25–80% of women taking AIs[4, 5]. Detrimental effects of AI-induced arthropathy (AIIA) include diminished quality of life, reduced adherence, and even discontinuation of treatment[6]. While nonsteroidal anti-inflammatory drugs (NSAIDs) may help relieve symptoms of joint arthropathy, such medications are not uniformly well-tolerated due to gastrointestinal and renal toxicity. Two recent randomized clinical trials showed reductions in AIIA with (1) duloxetine versus placebo at 12 weeks[7] and (2) acupuncture versus sham/wait list control at 6 weeks[8]. Despite the reported reduction in AI-induced joint symptoms, the side effects, possible drug interactions (duloxetine), high cost, and limited clinical acupuncture access may hinder the broad uptake of such interventions. Natural products such as omega-3 fatty acids have also been evaluated for treatment of AIIA[9], with post hoc analyses showing improved symptoms in obese versus non-obese women[10].

A naturally occurring polyphenol derived from turmeric root (*Curcuma longa*), curcumin is a widely used spice in Asian and Middle Eastern food. Turmeric also has a long history of medicinal use in China and India. As the bioactive ingredient of turmeric, curcumin is highly investigated as a natural product with potent anti-inflammatory and antioxidant properties[11, 12].

Several clinical trials support a potential role for curcumin in management of joint pain and stiffness. A registry study of Meriva^®^, a phosphatidylcholine complex of curcumin, showed improved walking distance in osteoarthritis patients with mild to moderate knee pain inadequately controlled by NSAIDs[13]. In a recent meta-analysis of randomized clinical trials of curcumin/turmeric for osteoarthritis or rheumatoid arthritis symptoms, 7 studies reported reduced pain and arthritis symptoms with curcumin/turmeric[14]. Clinical trials of curcumin have also indicated improvement in other conditions associated with AI therapy, such as depression/depressive symptoms[15–17], cognitive deficits[18, 19], and dyslipidemia[20].

The limited oral bioavailability (< 2%) and rapid hepatic metabolism of curcumin have posed obstacles to its clinical use. Development of novel nutraceutical delivery systems to enhance absorption and bioavailability of curcumin has included nanoemulsifying drug delivery systems using an isotropic mixture of surfactant and drug[21]. The surfactant-induced membrane fluidity and permeability changes improve drug bioavailability by enhancing solubilization, absorption, and protection against enzymatic hydrolysis[22, 23]. As an ideal vehicle for hydrophobic compounds such as curcumin, we developed a novel nanoemulsifying curcumin (NEC) that delivers much higher plasma levels of curcumin and its major metabolite curcumin-O-glucuronide in mice compared to suspension curcumin[23].

Taken together, these data led us to hypothesize that curcumin administered as a nanoemulsion might alleviate symptoms resulting from AI therapy and enable completion of the recommended treatment. We conducted a randomized double-blind pilot trial of NEC vs placebo in postmenopausal women with AIIA ≥ 3 months to determine feasibility of detecting changes in patient-reported outcome measures (PROMs), grip strength, plasma curcumin levels, and estrogen biomarkers following curcumin exposure.

## Methods

### Study oversight

This was a multi-institutional randomized, double-blind, placebo-controlled pilot study of NEC approved by the institutional review boards of City of Hope and The Ohio State University. The trial was conducted in accordance with institutional guidelines. Enrollment opened 4/2019 and closed 1/2021. Study participants were informed of the investigational nature of the study and provided written informed consent.

### Patient eligibility criteria

Eligible participants were postmenopausal women with stage I-IIIa ER- and/or PR-positive BC who reported at least 3 months or worsening of joint pain resulting from ongoing adjuvant AI therapy. Eligibility requirements included AIIA with score of ≥ 4, based on ≥ 3[24, 25] to ≥ 5[9] thresholds in prior AIIA trials, for the worst pain question of BPI-SF (0 for ‘no pain’ to 10 for ‘pain as bad as can imagine’). Exclusion criteria included: other prior invasive malignancy ≤ 5 years except treated non-melanoma skin cancer; history of anemia; bone fracture or surgery of affected joints < 180 days; medical therapy, alternative therapy, or physical therapy for joint symptoms ≤ 30 days or interarticular steroids ≤ 90 days; use of analgesics other than NSAIDs, acetaminophen < 14 days of study entry; use of medications that induce/inhibit CYP3A4; use of herbal/dietary supplements containing curcumin/curcuminoids ≤ 3 months of study entry; known sensitivity or allergy to turmeric or curry.

### Study intervention

Following signed informed consent, study participants were scheduled for the baseline visit (month 0) and randomized 1:1 to NEC or placebo. Only the pharmacist who dispensed the study agent was aware of randomization assignment. Women were advised to take 1 capsule twice daily for 3 months to provide either 200 mg/d of curcumin or placebo. Study capsules for NEC (lot #6103498) and matching placebo (lot #6103497) were manufactured by Lonza, Greenwood, SC. Each active capsule contained approximately 100 mg curcuminoids (Curcumin C3-complex^®^, Sabinsa, East Windsor, NH). Placebo capsules contained the same excipients, with food-safe dyes mixed to match the color and appearance of NEC-containing capsules.

Participants were instructed to complete daily logs of study capsule doses taken, use of analgesics, change in medications, and adverse events/symptoms. Daily logs and pill counts were reviewed monthly. Anthropometric measurements were taken at months 0 (T0) and 3 (T3). Grip strength was quantitated by hand dynamometer (JAMAR^®^, JLW instruments, Chicago, IL; Trailite Digital Hand dynamometer, Zhongshan, China) at T0 and T3; two trials were taken with each hand via squeezing the dynamometer for 2–3 s and releasing with a period of one-minute rest between trials. Blood samples were collected at T0 and T3 for complete blood count (CBC) and alanine aminotransferase (ALT) by institutional clinical laboratories. Plasma/serum aliquots were stored at − 80 °C for curcumin, estrone (E1) and estradiol (E2) levels for future batch analysis.

### PROMs

We used three tools to assess AIIA: (1) Functional Assessment of Cancer Therapy-Endocrine Symptoms (FACT-ES) measures physical, social and family, emotional, and functional well-being and endocrine symptoms with five response levels scored 0–4[26]; (2) Disabilities of the Arm, Shoulder and Hand (DASH) measures upper extremity physical function and joint symptoms, with 30 items scored 1–5 for health status over the past week regarding the degree of difficulty performing different physical activities due to arm, shoulder, and hand problems, severity of symptoms of pain, activity-related pain, tingling, weakness, and stiffness, and impact on social functioning, work, sleep, and self-image[27]; and (3) Brief Pain Inventory Short Form (BPI-SF) measures pain severity and pain interference in the last 24 h on 0–10 scales[28]. FACT-ES and DASH questionnaires were completed T0 and T3. BPI-SF scores were obtained T0, T1, T2, and T3.

### Curcumin and estrogen analyses

Plasma curcumin levels were determined by direct and indirect enzymatic assays at T0 and T3 using a validated liquid chromatography-tandem mass spectrometry (LC-MS/MS) method[29]. For the indirect enzymatic assay, plasma samples underwent ß-glucuronidase hydrolysis of curcumin glucuronide by method of Vareed et al.[30] prior to LC-MS/MS analysis of curcumin.

An ultrasensitive LC-MS/MS method in the clinically relevant sub-picomolar range for E1 and E2 was developed and validated by procedure of Bertelsen et al.[31]. Detailed sample preparation procedures and LC–MS/MS analytical settings and operating modes are provided in Supplementary Information for quantitative curcumin, E1 and E2 analyses.

### Statistical analysis

The primary goal of this pilot study was to evaluate the feasibility of using three validated instruments for evaluation of joint and other treatment-related symptoms in women on AI therapy taking NEC vs placebo in a randomized double-blind placebo-controlled trial. Sample size of 20 per group produces a two-sided 95% confidence interval with distance of 0.64 from the difference in means to the limits assuming a standard deviation (SD) of 1.0. Generalized Estimating Equation (GEE) was used to analyze the serially measured data for FACT-ES, DASH, BPI-SF using the compound symmetry working covariance matrix to account for within-person correlations across time and treating time as categorical. The interaction of treatment group by time was included in the GEE to test for differences in treatment effect over time. All randomized participants with at least one measurement were included in the GEE analysis.

For participants with measurements at T0 and T3, we calculated change scores (differences) between T3 and T0 (T3-T0) for FACT-ES, DASH, and BPI-SF and used the two-sample t-test to compare treatments. Following Hershman et al.[8], BPI-SF measurements at T1, T2, and T3 were considered clinically improved compared with T0 measurement for differences of ≥ 2 points. To compare the proportion of patients showing improvement across time between treatments, GEE for binary outcome using a compound symmetry working covariance matrix was used. Analyses used SAS 9.4.

Grip strength measurements for the two groups were compared using the two-sample t-test. Adherence was calculated by dividing the number of pills taken for days on study treatment by the number of pills dispensed for the days on study treatment.

## Results

### Study participants

**Fig. 1:**
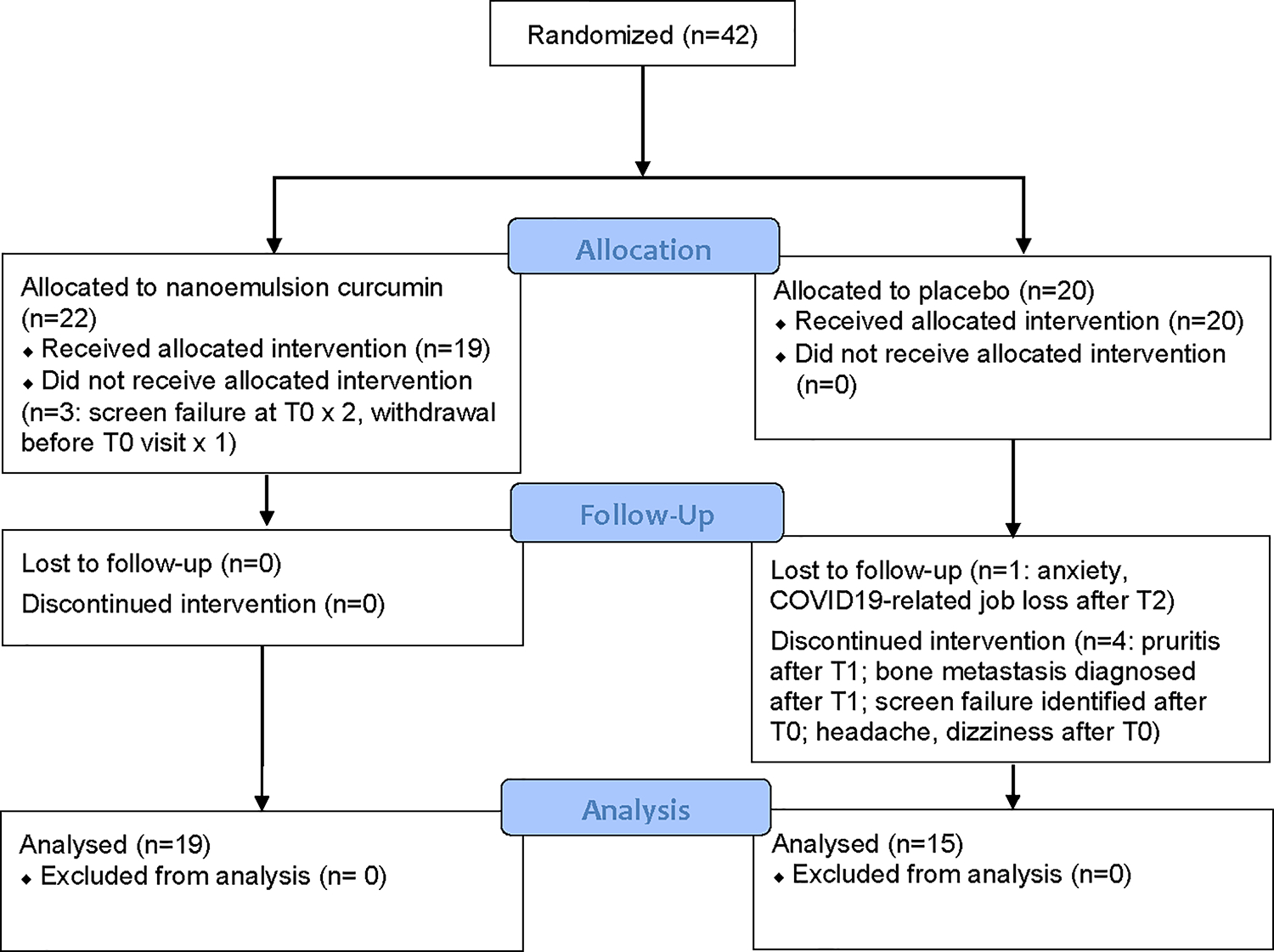
CONSORT flow diagram

From 4/2019 to 1/2021, 42 eligible postmenopausal women with ER positive BC and AIIA were randomized to NEC or matched placebo capsules for the 3-month study (Fig. 1). There were eight dropouts following enrollment/randomization (Fig. 1), resulting in 34 (81% of 42) participants (n = 15 placebo, n = 19 NEC) with evaluable measurements at both baseline and 3-month timepoints (81% total enrolled, 87% T0 participants). Table 1 provides the demographic details of the 42 randomized participants.

### Adherence

Based on pill counts and daily logs, average adherence to NEC/placebo for the 3-month study period was 92 ± 8.8% (n = 33 participants with one or both measures of adherence) and 90 ± 11.4% and 94 ± 5.8% in the placebo and NEC groups, respectively.

### Adverse events

No clinically significant changes were observed from T0 to T3 for CBC and ALT (n = 32). Common grade 1 adverse events (AE) reported by patients included diarrhea, nausea, heartburn, headache, stool discoloration, flatulence, abdominal pain, constipation, dyspepsia, and belching (Table 2). A total of six patients reported grade 2 AE that included headache, heartburn, and dyspepsia. One participant was diagnosed with bone metastasis; this serious AE was not attributed to the study intervention.

### PROMs

Of 42 participants enrolled, 39 initiated NEC/placebo with PROMs at T0 (93%) and 34/39 at both T0-T3 (87%).

#### FACT-ES

**Fig. 2:**
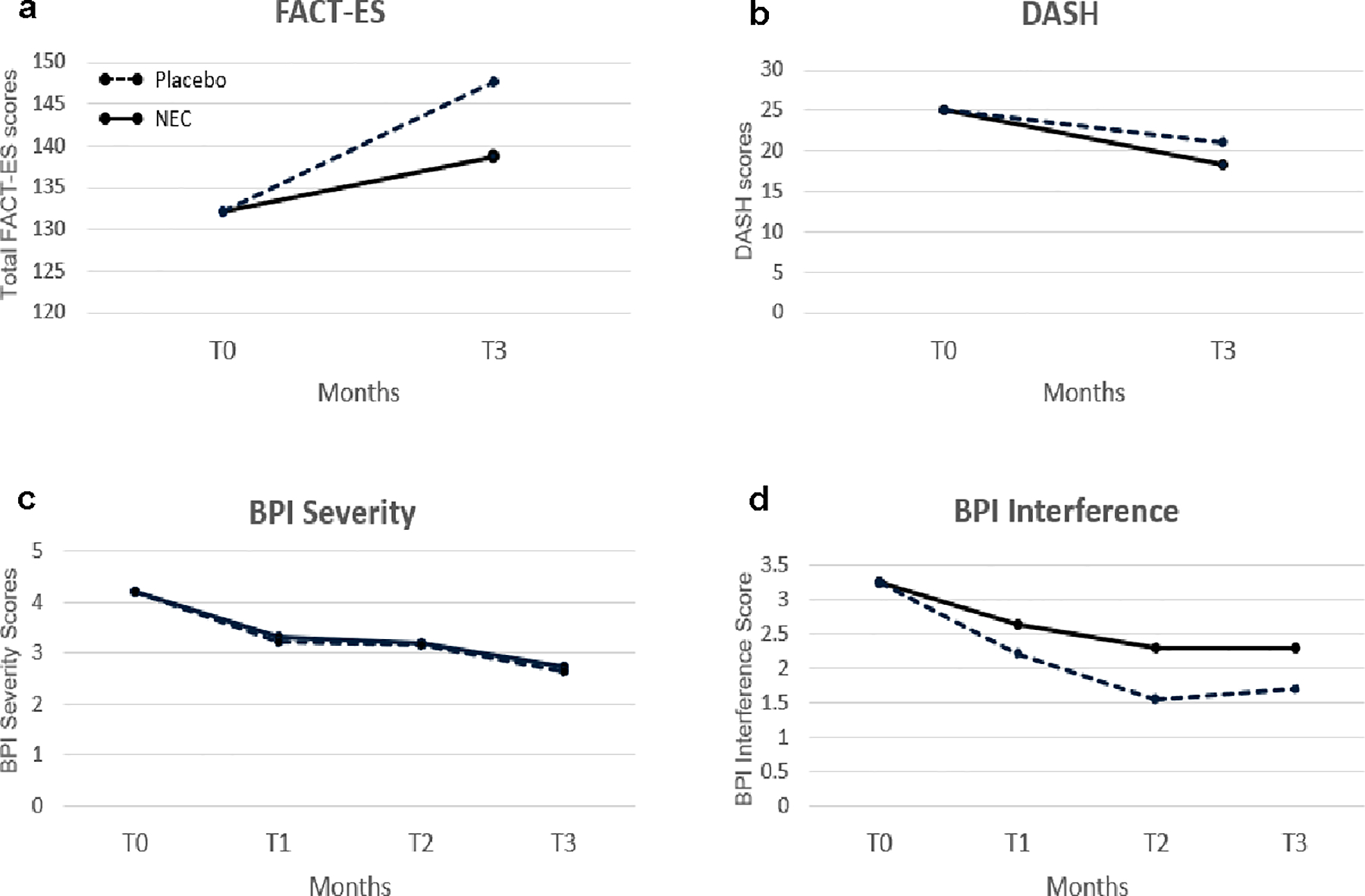
**a**–**d** Longitudinal analysis of FACT-ES, DASH, BPI severity, and BPI interference scores by GEE

Higher FACT-ES scores indicate better well-being and fewer symptoms. Treatment effect was significant in GEE analysis (p = 0.039), with higher FACT-ES scores in the placebo group compared to NEC group at T3 (Fig. 2a, Table 3). Although FACT-ES subscale scores for physical and social/family well-being and endocrine symptoms did not change significantly by treatment, scores for emotional well-being (p = 0.03) and functional well-being (p = 0.04) significantly increased in the placebo relative to NEC groups (Table 3).

For the 34 participants (n = 19 NEC, n = 15 placebo) with evaluable data at both T0 and T3, mean total FACT-ES scores (Table 4) at T0 did not significantly differ by study arm (placebo mean (M) = 135.8, SD = 19.8 vs NEC M = 127.4, SD = 21.8, p = 0.25) by two-sample t-test. However, the placebo versus NEC group had a significantly higher mean score at T3 (placebo M = 150.9, SD = 20.2, NEC M = 135.6, SD = 20.5; p = 0.038).

The average change scores of FACT-ES did not differ significantly by treatment arms (p = 0.11, Table 4). The mean FACT-ES change score showed an improvement from T0 to T3 in both study arms, which was significant in the placebo group (Mean change = 15.0, SD = 12.3, p < 0.01) but not in the NEC group (Mean change = 7.1, SD = 15.6, p = 0.08). FACT-ES change scores for the individual subscales did not differ significantly by study arm (data not shown).

#### DASH

A higher DASH score indicates a greater symptom burden. Treatment difference was not significant in GEE analysis (p = 0.43) (Fig. 2b, Table 3). For the thirty participants (placebo n = 13, NEC n = 17) with evaluable data at T0 and T3, mean baseline DASH scores were 20.6 (SD = 15.4) and 24.2 (SD = 17.4) for the placebo and NEC groups, respectively (p = 0.74). A comparison of the mean change scores by treatment arm did not show a significant difference (p = 0.42, Table 4). Symptom burden did not change between T3 and T0 for participants in NEC (mean change = −6.9, SD = 14.05, n = 17, p = 0.06) or placebo (mean change = −3.3, SD = 8.7, n = 13, p = 0.20) groups.

#### BPI-SF

The BPI-SF measures pain intensity (severity) and the impact of pain on functioning (interference) for the past 24 h. Pain severity is measured via four items (pain at its worst, pain at its least, pain on the average, pain right now) and interference by how much pain has disrupted seven daily activities (general activity, walking, work, mood, enjoyment of life, relations with others, sleep). Treatment difference over time for BPI severity as a composite of the four pain items (mean severity score) was not significant in GEE analysis (p = 1.0) (Fig. 2c, Table 3). Similarly, BPI interference as a mean of the seven interference items did not differ significantly by treatment arm in GEE analysis (Fig. 2d, Table 3).

For those with data at T0 and T3, mean T0 BPI severity scores were 3.8 (SD = 1.7, n = 15) and 4.2 (SD = 1.8, n = 19) for placebo and NEC groups, respectively (Table 4). For these women, mean BPI severity scores decreased significantly from T0 to T3 for NEC (mean change score of −1.5, SD = 2.1, n = 19, p < 0.01) and for placebo (mean change score of −1.4, SD = 1.9, n = 15, p = 0.01). The mean change scores between T3 and T0 did not differ significantly between study arms (p = 0.88).

For participants with T0 and T3 data, mean baseline BPI interference scores were 3.1 (SD = 2.4, n = 15) and 3.3 (SD = 1.9, n = 19) for placebo and NEC groups, respectively (Table 4). Mean BPI Interference scores decreased significantly from T0 to T3 in the placebo arm (mean change score of − 1.5, SD = 1.3, p < 0.01) but not in the NEC arm (mean change score of − 1.0, SD = 2.2, p = 0.07). The mean change scores between T3 and T0 did not differ significantly between study arms (p = 0.37).

**Fig. 3:**
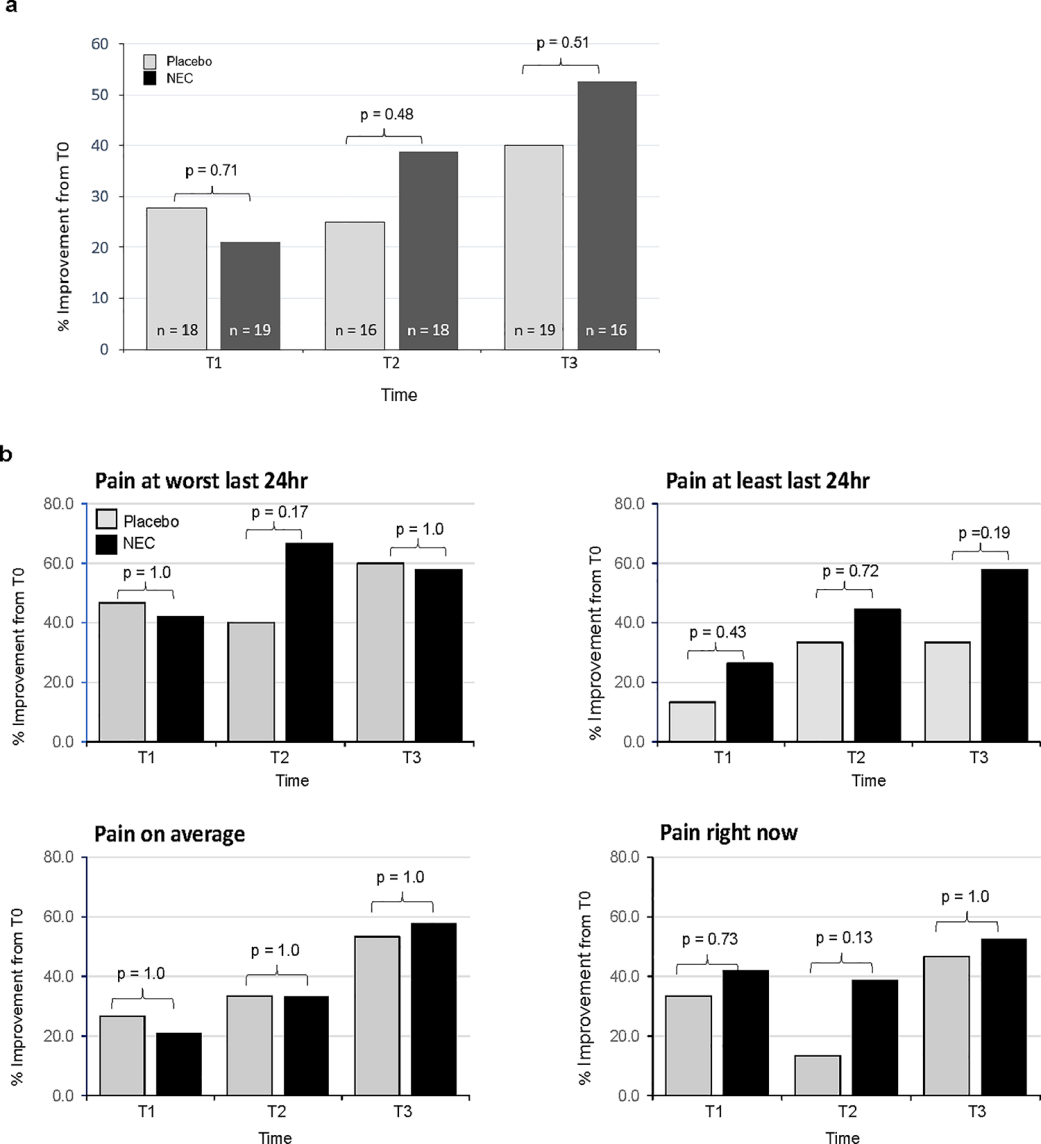
≥ 2-point improvement in BPI severity by **a** total and **b** component scores

Using a positive two-point change in BPI score as the threshold for clinically meaningful improvement[8], the proportion of women with ≥ 2-point improvement at T1, T2, T3 compared to T0 did not change significantly for BPI severity (p = 0.33) or BPI interference (p = 0.08); no treatment group differences in trends were found (p > 0.5) for BPI severity and interference. Nevertheless, we noted a nonsignificant increase in the percentage of participants with improvement in BPI severity at T2, and T3 compared to T0 for the NEC compared to placebo study group (Fig. 3a). Examining improvement in terms of the individual items of the BPI Severity scoring, scores for *pain at its least* within the last 24 h followed a nonsignificant increasing trend in the percent of patients with improvement at T1 to T3 (Fig. 3b).

Of the participants who completed the 3-month intervention, 10 of 19 (53%) participants in the NEC group compared to 6 of 15 (40%) in the placebo arm had BPI severity scores improved by ≥ 2 points (p = 0.51). For BPI Interference, 7 of 19 (37%) and 7 of 15 (47%) participants in the NEC and placebo groups, respectively, had ≥ 2 point improvement (p = 0.73).

### Grip strength

**Fig. 4:**
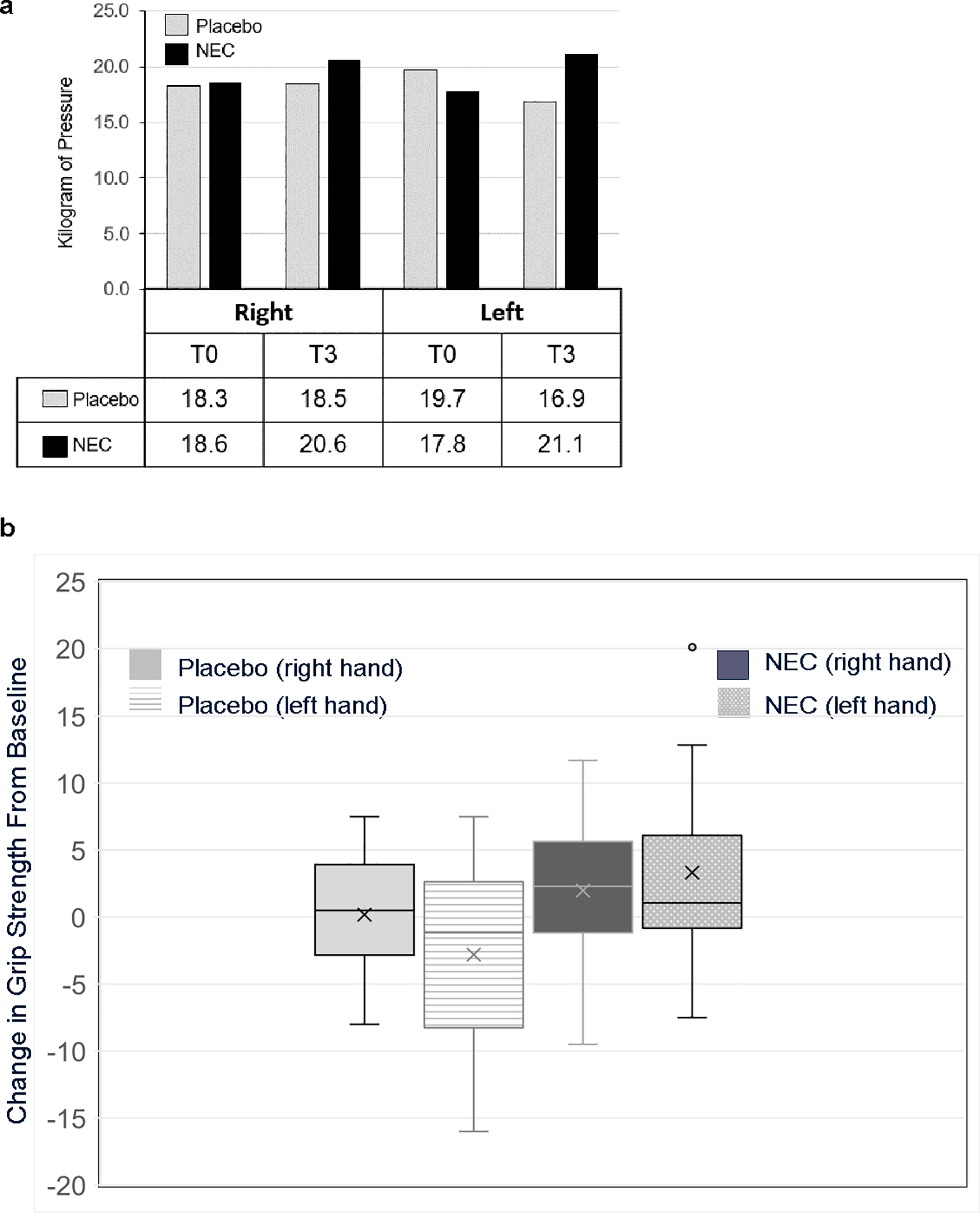
**a** Mean grip strength (kg) at T0 and T3 **b** Mean grip strength change T3–T0

Twenty-four participants underwent grip strength testing at T0 and T3; measurements obtained with the same dynamometer (Jamar or Trialite) at both timepoints were analyzed (n = 21). Baseline mean grip strength measurements for right and left hands were 18.6 (SD = 5.8) kg and 17.8 (SD = 6.5) kg, respectively, for NEC (n = 12) and 18.3 (SD = 4.7) kg and 19.7 (SD = 8.1) kg, respectively, for placebo (n = 9). At T3, average right and left grip strength measurements were 20.6 (SD = 6.8) and 21.1 (SD = 8), respectively, for NEC and 18.5 (SD = 4.9) and 16.9 (SD = 6.1), respectively, for placebo ( Fig. 4a); there were no statistically significant differences by paired Student’s t-test within or between study groups (p > 0.14). T0–T3 changes in grip strength varied by individuals, with increased and decreased measurements in both groups (Fig. 4b), without significant differences by treatment arm (right p = 0.44, left p = 0.07). Combining results of left and right grip strength change, more participants had increases > 2 kg in the NEC versus placebo group (12 readings, n = 7 NEC vs 6 readings, n = 4 placebo, p = 0.05).

### Plasma curcumin

**Fig. 5:**
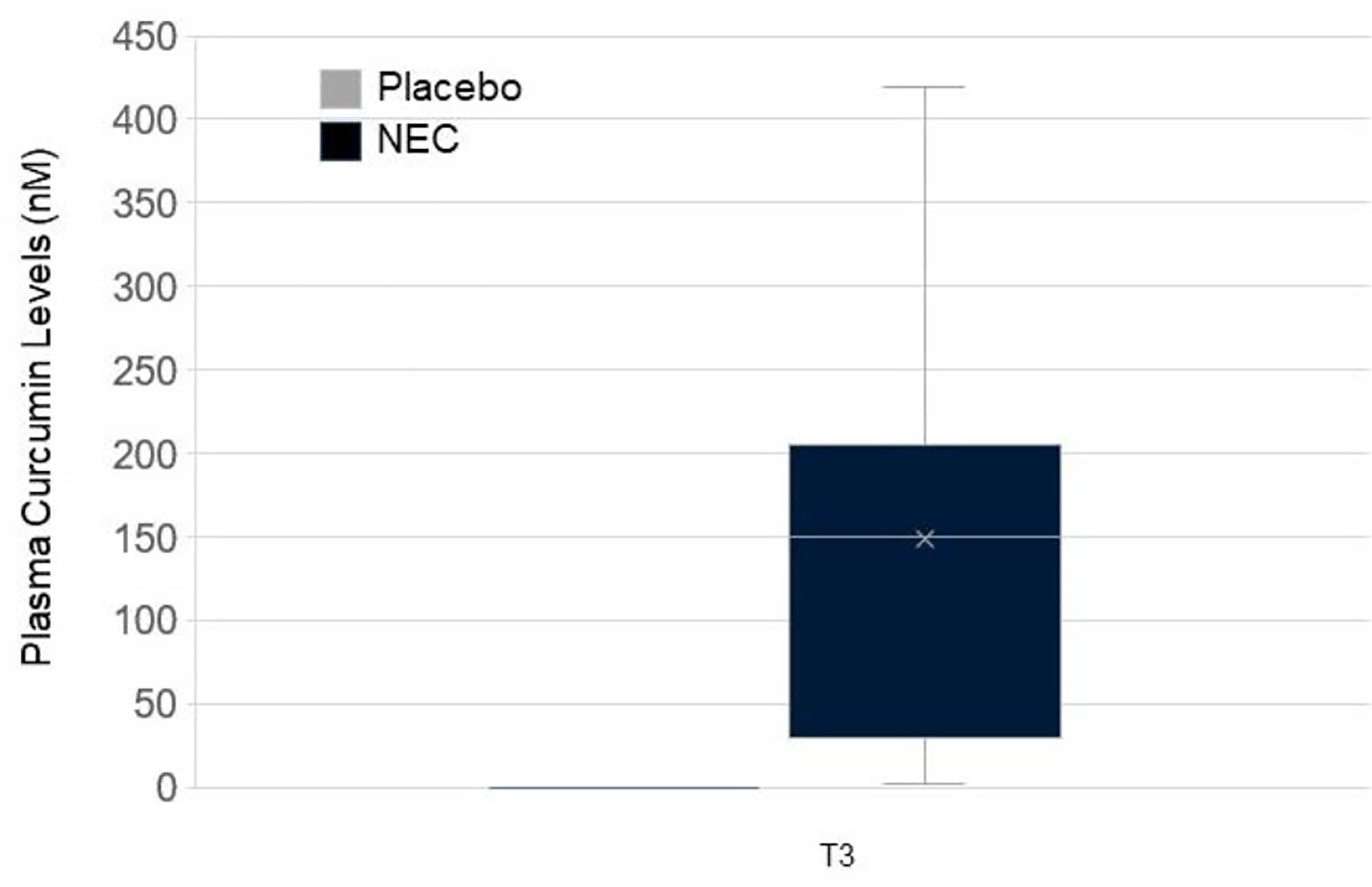
Quantitative determination of plasma curcumin

Blood samples were obtained from 32/34 T0-T3 participants. We determined curcumin plasma levels in participants with T3 samples obtained ≤ 2 days of the last dose of study agent (n = 14 NEC, n = 9 placebo). Curcumin was not detected in baseline plasma samples of either study group by direct or indirect methods. Following β-glucuronidase hydrolysis, curcumin was detected in all NEC samples at T3 from 2.56 to 419 nM, with an average level of 148 ± 127.1 nM (Fig. 5). None of the study participants in the placebo group had detectable T3 plasma curcumin following β-glucuronidase hydrolysis (Fig. 5). Analysis of study capsules (3 each) two years following manufacture confirmed curcumin content at the expected levels (data not shown).

### Serum E1 and E2

Serum E1 levels remained unchanged or below the lower limit of quantitation (LLoQ) at T0 and T3 in both study arms (n = 14 NEC, n = 9 placebo). Serum E2 decreased in two placebo arm patients, from 302.9 pmol/L and 107.6 pmol/L at baseline, respectively, to < LLoQ (< 3.67 pmol/L) at T3; E2 was otherwise unchanged < LLoQ from T0-T3.

## Discussion

With this first randomized controlled, double-blind pilot trial of curcumin in postmenopausal women experiencing AIIA, we demonstrated feasibility of this multi-site study design by the following measures: (1) 87% completion of PROMs at T0-T3 (34/39 participants with T0); (2) 82% collection of lab/biospecimens at T0-T3 (32/39 participants with T0); (3) adherence > 90% in both arms; (4) accrual in 20 months with 87% T0 visits within 11 months until March 2020 and ensuing COVID19 pandemic restrictions on in-person clinical encounters.

Both study agents were well-tolerated, without attributable serious AEs. Gastrointestinal symptoms were the most common AE, which is consistent with reports of other trials using a variety of different curcumin formulations[18].

While this pilot trial was not powered to test for efficacy, we conducted analyses for preliminary estimates of differences between the two treatments. For the 34 participants with paired 0–3-month assessments, changes in PROM scores at T3 did not differ significantly between NEC and placebo; both arms had higher scores at T3 vs baseline. No adjustments were made for multiple tests for this pilot study; the p-value (p = 0.039) for FACT-ES did not account for multiple tests. As the placebo contained only excipients/food dyes, the greater improvement in FACT-ES and BPI-Interference scores in women taking placebo vs NEC was unexpected (Table 3). Increasing public awareness of the potential benefits of curcumin/turmeric based on traditional and Ayurvedic medicine contributed to patient interest in this trial and might have affected the reported outcomes with placebo[32].

We observed that BPI-Severity scores for the last 24 h, using BPI-SF, might not accurately capture the experience of joint arthropathy for our patient cohort where most women described waxing and waning symptoms with good and bad days. Notably, we observed a non-significant trend for the BPI severity component of *least pain in last 24 h* when analyzing for ≥ 2 point improvement. A more nuanced assessment of pain with BPI Long Form (e.g., pain over a week) or Penn Arthralgia score[33] might refine our ability to evaluate AIIA and treatment efficacy in future studies.

By grip strength, we noted a trend toward improvement with NEC relative to placebo treatment. The number of participants with pre- and post-intervention dynamometer readings was limited by the COVID19 pandemic, with decreased clinic access and patient preferences to avoid in-person follow-up in 2020 and 2021.

For this pilot project, plasma curcumin by enzymatic hydrolysis provided a biomarker of exposure, detected only in the NEC group samples. Most curcumin pharmacokinetic studies involve hydrolyzing plasma samples prior to quantitation due to the rapid metabolism of curcumin. Currently, there are no published pharmacokinetic studies evaluating the drug-drug interaction of curcumin and AIs. Our preliminary data on the lack of increased E1 and E2 serum levels suggest that curcumin may not enhance AI metabolism. We plan to correlate optimal curcumin dose with treatment effects in future studies.

Randomized controlled trials have demonstrated beneficial effects of curcumin for a wide range of clinical indications, including reduced osteoarthritis pain[13, 34, 35], improved memory and attention[19], decreased depressive symptoms[15], and anti-diabetic[36] and lipid-lowering[20] properties. Our limited study sample size was not powered for efficacy. Higher doses and longer duration of treatment with NEC might also be needed for amelioration of AIIA, despite reports of short-term improvement in osteoarthritis patients treated with curcumin[35, 37]. In addition, the COVID19 pandemic contributed to the lower completion rate of certain study endpoints, such as blood draws and grip strength measurements.

Taken together, this pilot study demonstrated the feasibility of a randomized double-blind, placebo-controlled trial of curcumin in women experiencing AIIA. We observed potential trends for improvements specific to NEC vs placebo in BPI-SF severity subscores and grip strength, with plasma curcumin as a viable marker of exposure. These study results provide the foundation for a future clinical trial to evaluate NEC for efficacy and safety in ameliorating AIIA.

## Supplementary Material

Lustberg_Yee_BCRT_Supplementary_Material

## Figures and Tables

**Table 1:** Demographics and Disease characteristics, baseline

Variable	NEC (n=22) N (% arm) or Median (Range)	Placebo (n=20) N (% arm) or Median (Range)	All (n=42) N (% all) or Median (Range)

Age (years)	61 (42 – 71)	58 (39 – 72)	60 (39 – 72)

Race			
Caucasian	18 (82)	17 (85)	35 (83)
Asian	2 (9)	2 (10)	4 (10)
Black	2 (9)	0 (0)	2 (5)
Unknown	0 (0)	1 (5)	1 (2)

Ethnicity			
Hispanic	4 (18)	6 (30)	10 (24)
Non-Hispanic	18 (82)	14 (70)	32 (76)

Body Mass Index	26.5 (19.8 – 36.6) N=20	29.9 (21.3 – 42.0) N=19	28.3 (19.8 – 42.0) N=39

TNM Staging			
Stage IA	4 (18)	9 (45)	13 (31)
Stage IIA, B	15 (68)	10 (50)	25 (59)
Stage IIIA	3 (14)	1 (5)	4 (10)

Progesterone Receptor			
Positive	17 (77)	19 (95)	36 (86)
Negative	5 (23)	1 (5)	6 (14)

HER2/neu			
Negative	20 (91)	16 (80)	36 (86)
Positive	2 (9)	4 (20)	6 (14)

Chemotherapy			
None	8 (36)	13 (65)	21 (50)
Adjuvant	9 (41)	6 (30)	15 (36)
Neoadjuvant	3 (14)	1 (5)	4 (10)
Both	2 (9)	0 (0)	2 (5)

Radiation Therapy (RT)			
None	7 (32)	4 (20)	11 (26)
Partial Breast	1 (5)	3 (15)	4 (10)
Post Mastectomy	5 (23)	8 (40)	13 (31)
Whole Breast	8 (36)	4 (20)	12 (28)
RT but not specified	1 (5)	1 (5)	2 (5)

**Table 2:** Common adverse events by grade and treatment group

Adverse Events by Grade	Number of patients (%)	

	NEC (n=19)	Placebo (n=20)

Grade 1		
Diarrhea	0	2 (10.0)
Nausea	2 (10.5)	1 ( 5.0)
Heartburn	3 (15.8)	3 (15.0)
Headache	3 (15.8)	6 (30.0)
Stool discoloration	2 (10.5)	0
Flatulence	3 (15.8)	7 (35.0)
Abdominal pain	2 (10.5)	1 (5.0)
Constipation	3 (15.8)	0
Dyspepsia	2 (10.5)	2 (10.0)
Belching	0	1 (5.0)

Grade 2		
Headache	1 (5.3)	2 (10.0)
Heartburn	0	1 (5.0)
Dyspepsia	1 (5.3)	1 (5.0)

**Table 3:** Summary of FACT-ES, DASH, and BPI-SF scores^a^

	**NEC**						**Placebo**						**p-value between arms** **
**FACT-ES, total**	**N**	**Mean**	**SD**	**Median**	**Min**	**Max**	**N**	**Mean**	**SD**	**Median**	**Min**	**Max**	
**Baseline**	20	130.2	22.4	130.9	91.0	163.0	20	133.9	21.3	135.0	94.9	172.0	
**3 months**	19	137.0	22.2	138.8	101.0	173.0	15	150.9	20.2	148.6	113.0	175.0	
**p-value within arm** *	0.045						<.0001						0.039
FACT-ES, PWB	**N**	**Mean**	**SD**	**Median**	**Min**	**Max**	**N**	**Mean**	**SD**	**Median**	**Min**	**Max**	
**Baseline**	20	19	6.2	21	7	27	20	19	5.4	18.5	9	27	
**3 months**	19	21.7	4.4	23	8.2	28	15	22.9	5.0	25	12	28	
**p-value within arm** *	0.026						<.0001						0.58
FACT-ES. SWB	**N**	**Mean**	**SD**	**Median**	**Min**	**Max**	**N**	**Mean**	**SD**	**Median**	**Min**	**Max**	
**Baseline**	20	21.9	4.7	21.5	11.7	28	20	24	3.7	24	15.7	28	
**3 months**	19	21.7	5.8	23	8.2	28	15	24.9	4.3	27	12	28	
**p-value within arm** *	0.68						0.042						0.16
FACT-ES, EWB	**N**	**Mean**	**SD**	**Median**	**Min**	**Max**	**N**	**Mean**	**SD**	**Median**	**Min**	**Max**	
**Baseline**	20	18.8	4.1	20	11	28	20	19	2.8	19	11	23	
**3 months**	19	18.6	4.8	20	8	24	15	20.7	2.3	20	17	24	
**p-value within arm** *	0.89						0.0004						0.03
FACT-ES, FWB	**N**	**Mean**	**SD**	**Median**	**Min**	**Max**	**N**	**Mean**	**SD**	**Median**	**Min**	**Max**	
**Baseline**	20	20.4	5.4	21.1	9	28	20	20.2	5.6	21.5	7	27	
**3 months**	19	20.1	6.5	22	7	28	15	21.6	5.9	23	10.5	28	
**p-value within arm** *	0.83						0.007						0.04
FACT-ES, ESS-19	**N**	**Mean**	**SD**	**Median**	**Min**	**Max**	**N**	**Mean**	**SD**	**Median**	**Min**	**Max**	
**Baseline**	20	50.5	15.7	51.4	0	69	20	51.1	10.8	51	31	70	
**3 months**	19	54.9	16.7	58	0	72	15	60.7	7.7	59	48	71	
**p-value within arm** *	0.0012						<0.0001						0.16
**DASH**	**N**	**Mean**	**SD**	**Median**	**Min**	**Max**	**N**	**Mean**	**SD**	**Median**	**Min**	**Max**	0.43
**Baseline**	19	23.9	17.3	24.2	0.0	63.3	19	25.7	16.4	24.2	3.3	55.4	
**3 months**	18	17.6	17.5	13.8	0.0	57.8	14	18.1	17.6	16.2	0.0	55.8	
**p-value within arm** *	0.039						0.15						
**BPI-SF, Severity**	**N**	**Mean**	**SD**	**Median**	**Min**	**Max**	**N**	**Mean**	**SD**	**Median**	**Min**	**Max**	1.0
**Baseline**	20	4.1	1.7	3.8	1.3	7.0	20	4.3	1.8	4.8	1.0	7.3	
**1 month**	19	3.3	1.5	3.0	1.5	6.8	18	3.2	1.6	2.8	1.0	5.8	
**2 months**	18	3.2	2.2	2.5	0.0	7.3	16	3.0	1.8	2.6	0.0	6.3	
**3 months**	19	2.7	2.0	2.3	0.0	8.5	15	2.5	1.8	2.3	0.0	5.8	
**p-value within arm** *	0.009						0.0003						
**BPI-SF, Interference**	**N**	**Mean**	**SD**	**Median**	**Min**	**Max**	**N**	**Mean**	**SD**	**Median**	**Min**	**Max**	0.6
**Baseline**	20	3.3	1.9	3.2	0.0	6.9	20	3.2	2.1	3.1	0.1	7.6	
**1 month**	19	2.7	2.0	2.4	0.0	6.7	18	2.2	2.0	1.6	0.0	5.9	
**2 months**	18	2.4	2.2	2.1	0.0	7.1	16	1.4	1.6	1.1	0.0	5.9	
**3 months**	19	2.3	2.4	2.1	0.0	9.0	15	1.6	1.8	0.9	0.0	4.9	
**p-value within arm** *	0.26						<.0001						

a Functional Assessment of Cancer Therapy-Endocrine Symptoms (FACT-ES): Total is the sum of the physical well-being (PWB), social/family well-being (SWB), emotional well-being (EWB), functional well-being (FWB), and endocrine subscale (ESS-19); Disabilities of the Arm, Shoulder, and Hand (DASH); Brief Pain Inventory-Short Form (BPI-SF)

* p-values for T0 to T3 comparison within arm by generalized estimating equations (GEE)

** p-values for NEC vs placebo by GEE

**Table 4:** FACT-ES, DASH, and BPI-SF scores^a^ and change from 0 to 3 months (T3-T0 change scores)

	**NEC (N=19)**						**Placebo (N=15)**						**p-value between arms** **
**FACT-ES, total**	**N**	**Mean**	**SD**	**Median**	**Min**	**Max**	**N**	**Mean**	**SD**	**Median**	**Min**	**Max**	
**Baseline**	19	127.4	21.8	127.0	91.0	162.0	15	135.8	19.8	135.0	110.0	172.0	
**3 months**	19	135.6	20.5	138.8	101.0	167.0	15	150.9	20.2	148.6	113.0	175.0	**0.11**
**Change Score**	19	7.1	15.6	7.8	−26.8	31.1	15	15.0	12.3	14.0	−7.4	33.0	
**p-value within arm** *	**0.08**						**<0.01**						
	**NEC (N=17)**						**Placebo (N=13)**						**0.42**
**DASH**	**N**	**Mean**	**SD**	**Median**	**Min**	**Max**	**N**	**Mean**	**SD**	**Median**	**Min**	**Max**	
**Baseline**	17	24.2	17.4	24.2	0.0	63.3	13	20.6	15.4	13.3	3.3	51.7	
**3 months**	17	17.3	18.0	12.1	0.0	57.8	13	17.3	18.0	14.2	0.0	55.8	
**Change Score**	17	−6.9	14.0	−1.2	−43.3	14.2	13	−3.3	8.7	−3.3	−24.2	10.8	
**p-value within arm** *	**0.06**						**0.20**						
	**NEC (N=19)**						**Placebo (N=15)**						**0.88**
**BPI-SF, Severity**	**N**	**Mean**	**SD**	**Median**	**Min**	**Max**	**N**	**Mean**	**SD**	**Median**	**Min**	**Max**	
**Baseline**	19	4.2	1.8	4.0	1.3	7.0	15	3.8	1.7	4.5	1.0	6.5	
**3 months**	19	2.7	2.0	2.3	0.0	8.5	15	2.5	1.8	2.3	0.0	5.8	
**Change Score**	19	−1.5	2.1	−2.5	−5.0	1.8	15	−1.4	1.9	−1.0	−4.5	1.0	
**p-value within arm** *	**<0.01**						**0.01**						
	**NEC (N=19)**						**Placebo (N=15)**						**0.37**
**BPI-SF, Interference**	**N**	**Mean**	**SD**	**Median**	**Min**	**Max**	**N**	**Mean**	**SD**	**Median**	**Min**	**Max**	
**Baseline**	19	3.3	1.9	3.4	0.0	6.9	15	3.1	2.4	3.1	0.1	7.6	
**3 months**	19	2.3	2.4	2.1	0.0	9.0	15	1.6	1.8	0.9	0.0	4.9	
**Change Score**	19	−1.0	2.2	−1.3	−5.6	3.4	15	−1.5	1.3	−1.1	−3.1	0.4	
**p-value within arm** *	**0.07**						**<0.01**						

a Functional Assessment of Cancer Therapy-Endocrine Symptoms (FACT-ES); Disabilities of the Arm, Shoulder, and Hand (DASH); Brief Pain Inventory-Short Form (BPI-SF)

* The p-values are based on the one-sample t-test to test if the mean change score within a group equals 0.

** The p-values are based on the two-sample t-test to test if the mean change scores between NEC and placebo are equal.

## Data Availability

The datasets generated and/or analyzed during the current study are not publicly available to protect study participant privacy.

## Abbreviations

AE: Adverse events
AIs: Aromatase inhibitors
AIIA: Aromatase inhibitor-induced arthralgia
ALT: Alanine aminotransferase
BC: Breast cancer
BPI-SF: Brief Pain Inventory short form
CBC: Complete blood count
DASH: Disabilities of the shoulder, arm, and hand
E1: Estrone
E2: Estradiol
ER: Estrogen receptor
FACT-ES: Functional assessment of cancer treatment-endocrine symptoms
GEE: Generalized estimation equation
LC–MS/MS: Liquid chromatography-tandem mass spectrometry
LLoQ: Lower limit of quantitation
M: Mean
NEC: Nanoemulsion curcumin
NSAIDs: Nonsteroidal anti-inflammatory drugs
PR: Progesterone receptor
PROMs: Patient-reported outcome measures
SD: Standard of deviation
