# Supplementary material for: Randomized placebo-controlled, double-blind clinical trial of nanoemulsion curcumin in women with aromatase inhibitor-induced arthropathy: an Alliance/NCORP pilot trial
Source: Breast Cancer Res Treat. Author manuscript; Available in PMC 2024 May 2. (PMC11062803; doi:10.1007/s10549-023-07223-4)
Supplement: Supplementary file 1 [file NIHMS1980028-supplement-Lustberg_Yee_BCRT_Supplementary_Material.doc]

Supplementary Information

Randomized placebo-controlled, double-blind clinical trial of nanoemulsion curcumin in women with aromatase inhibitor-induced arthropathy: an Alliance/NCORP pilot trial

Author information

Maryam Lustberg, Patty Fan-Havard, Lennie Wong, Kasey Hill, Mitch A. Phelps, Kevin W. Herrera, Ni-Chun Tsai, Timothy Synold, Ye Feng, Chidimma Kalu, Mina S. Sedrak, Lisa D. Yee

Corresponding Author:

Lisa D. Yee

City of Hope

lyee@coh.org

LC-MS/MS Assay Method for Curcumin

Curcumin, curcumin-d6 internal standard, and plasma curcumin levels in clinical samples were determined by direct and indirect enzymatic assays at 0 (baseline) and 3 months using a validated liquid chromatography-tandem mass spectrometry (LC-MS/MS) method.[21] Curcumin and curcumin-d6 internal standard were monitored using a TSQ Quantiva triple quadruple mass spectrometer with a heated electrospray ionization (HESI) source coupled with a Vanquish Ultra High Pressure Liquid Chromatograph (Thermo Fisher Scientific Inc., Waltham, MA).

The chromatographic separation was performed on a Zorbax Extend-C18 column at 30°C using gradient elution with 0.2% formic acid (v/v) and acetonitrile:isopropanol (8:2) with 0.2% formic acid (v/v). The total run time was 9 minutes. Curcumin and curcumin-d6 were measured by selected reaction monitoring (SRM) in positive polarity at m/z of 369.212 → 177.054 and 375.212 → 180.054, respectively. Both compounds used a collision energy of 20 V and the RF lens was set at 60 V. The mass spectrometer used argon as the collision gas at a pressure of 1.5 mTorr, an ion spray voltage of 3300 V, nitrogen sheath and auxiliary gas at 40 and 8 arbitrary units, an ion transfer tube temperature of 325°C, and a vaporizer temperature of 300°C. Curcumin concentration in human plasma was quantitated using isotope dilution response ratios.

Sample preparation A 100uL aliquot of plasma was mixed with curcumin-d6 internal standard and 50% acetonitrile, followed by the addition of *tert*-Butyl methyl ether and then vortexed and centrifuged at 13,500rpm for 10 minutes at 4˚C. The supernatant was transferred to a glass tube and evaporated under nitrogen, then reconstituted with 150ml 50% acetonitrile, vortexed, and centrifuged at 3900rpm for 5 minutes. For the indirect enzymatic assay, plasma samples were treated for the enzymatic hydrolysis of curcumin glucuronide according to the method of Vareed et al[23] by mixing with ß-glucuronidase from E. coli (Sigma Aldrich, St. Louis, MO: 125uL, 250 units) in 0.1 M phosphate buffer (pH 6.8) and incubating at 37°C for 3.5 hours before extraction and evaporation. The reconstituted supernatant samples were transferred to a 220 uL autosampler plate for LC-MS/MS analysis with a 5ml injection volume subjected to LC-MS/MS analysis.

Data processing Xcalibur software (version 4.4_16.14) was used for instrument control and data acquisition and processing.

LC-MS/MS Assay Method for Estrone (E1) and Estradiol (E2)

LC-MS/MS system consisted of a Shimadzu Prominence HPLC system interfaced to an AB SCIEX QTRAP® 5500 system (Foster City, CA, USA). HPLC separation was achieved using a Kinetex 1.7 um Phenyl-Hexyl 100x2.1 mm 1.7 um, (Phenomenex). The column temperature was maintained at 60ºC, and the flow rate was 0.38ml/min. The mobile phase consisted of A (Water: 1000ml + 60ul 30%NH4OH) and B (Methanol: 1000ml+ 60ul 30% NH4OH). The following gradient program was used: 55% B (0.01 min), 70% B (0.01-4.0 min), 100% B (5.5 min), 30%B (8.5 min). The total run time was 8.5 min. The auto-injector temperature was maintained at 15ºC. The atmospheric pressure chemical ionization (APCI) source of the mass spectrometer was operated in negative ion mode with ion source gas1 (55), curtain gas (20), collision gas (High), nebulizer current -4.0, The entrance potential was set to -10V. Declustering potential (DP) was -110, collision energy (CE), and collision cell exit potential (CXP) was optimized to -50V, -21V for E1, -160V, -50V, -17V for E1 internal standard, -210V, -58V, -19V for E2 and -205V, -52V, -13V for E2 internal standard respectively. The source temperature was 400ºC. A solvent delay program was used from 0 to 2.0 min and from 4.0 to 8.5 min to minimize the mobile phase flow to the source. Atmospheric pressure chemical. ionization of E1, E1 D4, E2, E2 D5 produced abundant protonated molecular ions (MH-) at m/z 268.980, 272.983, 270.969, and 275.981 respectively. Fragmentation of these compounds was induced under collision induced dissociation conditions. The precursor→product ion combinations at m/z 268.980→145.200 for E1, and 272.983→147.100 for E1 internal standard, 270.969→182.800 for E2, and 275.981→147.000 E2 internal standard were used in multiple-reaction monitoring mode for quantitation. Under optimized assay conditions, the retention times for E1, E1 internal standard and E2 and E2 internal standard were 3.04 and 3.35 min, respectively.

Sample preparation Estrone and estradiol standards ranging from 4 and 20 pg/ml to 200 and 1000 pg/ml and controls at 12 and 60, 60 and 300 and 160 and 800 pg/ml were prepared from stock solutions by dilution in 50% MeoH for working solution. Standards were prepared by spiking 50uL working solution into 450uL water. 500ul

aliquots for fresh standard, control and human serum sample spiked with 5ul 50% MeOH containing internal standards E1 D4 and E2 D5 at 1 and 5ng/ml, extracted with 1 ml Hexane:MTBE (75:25) , vortexed for 10 min, and added 75uL Hexane:IPA (75:25), then centrifuged for 5 min at 14,000g at 4ºC, the organic phase was transferred to a 1.5mL tube and evaporated to dryness at 40ºC under nitrogen. The residue was reconstituted with 50ul 50% MeOH, then transferred to 0.5ml microcentrifuge tube, centrifuge 5 min at 10,000 rpm, the contents were transferred to insert for LC/MS/MS analysis. A 25ul sample was injected.

Data processing Analyst software version 1.6.1 was used for data acquisition and processing.
